# Reassessing Breeding Investment in Birds: Class-Wide Analysis of Clutch Volume Reveals a Single Outlying Family

**DOI:** 10.1371/journal.pone.0117678

**Published:** 2015-01-29

**Authors:** David M. Watson, Susan E. Anderson, Valerie Olson, for the Institute for Land, Water and Society

**Affiliations:** 1 Charles Sturt University, Albury, New South Wales, Australia; 2 Zoological Society of London, London, England; Hungarian Academy of Sciences, HUNGARY

## Abstract

Reproductive investment is typically considered in terms of size and number of propagules produced. Compared with a thorough understanding of the overall patterns and ecological correlates of avian clutch size, egg size has received less attention and the total effort invested in laying a clutch of eggs is rarely considered. We used clutch volume as an alternative estimate of reproductive investment and present the first class-level analysis of clutch volume in birds using 1,364 randomly-selected species in 204 families. The relationship between body mass and egg volume was very strong (r^2^ = 0.946), validating previous studies identifying four families (Apterygidae, Pelecanoidiididae, Sternidae and Dromadidiae) with disproportionately large eggs. Clutch volume was also closely related to body mass (r^2^ = 0.909) and all but one of the taxa with disproportionately large eggs conformed to the overall relationship, their greater egg dimensions compensated by diminished clutch size. The only family which departed significantly from the relationship between body mass and clutch volume was the mound builders (Megapodiidae)—the only group of birds that do not rely on body heat for incubation. Although previously known for laying large clutches of large eggs containing disproportionately large yolks, the remarkable investment of megapodes in reproduction (more than seven times greater than other birds of comparable mass) has been hitherto overlooked. We consider the evolutionary basis and ecological implications of this finding, suggesting that energetic costs associated with incubation act as an upper limit on reproductive output of other birds. We recommend clutch volume as a sensitive, fine-grained measure of reproductive effort for research at a wide range of scales and advocate further analysis of ecological correlates of clutch volume in birds and amniotes generally.

## Introduction

“*Voyez-vous cet oeuf*. *C’est avec cela qu’on renverse toutes les écoles de théologie*, *et tous les temples de la terre*.” Diderot (Le rêve de d’Alembert 1769)

[“*See this egg*. *It is with this that all theological theories and all the temples of the earth are overturned*.”]

A defining characteristic of any organism is the effort devoted to reproduction, underpinning some of the founding principles in both evolution and ecology [1]. Most comparative research has focused on propagules (seeds, spores, eggs) and there is an established theoretical literature on the evolution of propagule size and number [2]–[4]. Eggs have been the focus of most empirical research on animal reproduction, in part due to the ease with which they can be counted and measured, and the use of egg size and number to quantify reproductive effort is commonplace [5]–[8]. Of all amniotes, birds exhibit the greatest variation in the number of eggs laid, from a single egg every two or three years by some albatrosses to more than 20 each year for some partridge and grouse species [9]. Building on the monographic research of Moreau [10] and Lack [11], [12], comparative biologists have explored the basis of this variation and there is now a voluminous literature on biogeographic, phylogenetic, autecological and environmental correlates of clutch size in birds [13]–[15].

As well as varying in clutch size, birds also exhibit considerable variation in egg size [16], [17], and combining these two measures provides a more inclusive measure of the energy expended in clutch creation than using either egg size or clutch size alone [18]. Unlike the integer-based clutch size, clutch volume is continuous and as such, provides a more finely resolved estimate of variation in reproductive effort. Several studies have used this variable for research on single species or within a family [19], [20] but few higher-level comparisons of clutch volume have been made (*e*.*g*., [17], [21]) and, to-date, the nature and extent of variation in clutch volume across all birds has escaped attention.

Here, we present the first comprehensive examination of variation in clutch volume across modern birds to reveal overall patterns and identify notable exceptions in breeding investment. Having evaluated the strength and shape of the relationship between body mass and clutch volume, we identify those groups that diverge from the overall allometric relationship and compare them with those taxa deemed exceptional in previous work based on egg dimensions (both mass and volume). Specifically, how does the clutch volume of those taxa with disproportionately large eggs compare with other birds? We consider whether clutch volume represents a viable complement to clutch size in terms of practicality, explanatory ability and biological relevance. We stress the importance of random taxon sampling when conducting higher-level comparisons and urge ornithologists to consider reproductive investment in a more holistic manner than simply the number of eggs laid. Using these data, we have also quantified the relationship between clutch volume and various life history traits, comparing emergent relationships with previous findings regarding correlates of clutch size, but these findings will be presented elsewhere.

## Methods

To prevent phylogenetic structure from influencing the overall relationship and artificially minimising variance estimates, family means (after [22]) were used instead of species-level data, consistent with previous work that recognized 80–90% of avian life history variation is fixed at the family scale [12], [23]. Several compilations of family life history traits are available but none contained egg dimension data. To evaluate existing databases for another trait (body mass), we conducted a trial with 15 families from 10 orders, encompassing speciose and species-poor families, and families with both wide and narrow variation in body mass (exhibiting sexual dimorphism and monomorphism): Anatidae, Phasianidae, Scolopacidae, Columbidae, Psittacidae, Cuculidae, Strigidae, Caprimulgidae, Alcedinidae, Cotingidae, Maluridae, Paridae, Paradisaeidae, Corvidae and Icteridae. For each family, body mass for all species for which data could be sourced (primarily from the primary literature and specimen data) were compiled and an overall mean and standard deviation of body mass calculated. These mean values were compared with means used by Bennett and Owens [24]; values were unavailable for six families, within 15% for six and greater than 20% different for three families. Their values for Phasianidae and Strigidae (996 and 600, respectively) were dramatically different to ours (408 and 183) and, in the absence of further information about sample sizes and selection criteria, we elected to compile our own database for body mass, egg dimensions and clutch size.

To determine the minimum number of species required to represent within-family variation accurately, a random sample of 20 species from each of these 15 families was selected and a mean for this sample calculated. Single sample t-tests with 100 iterations were repeated for every sample size from 20 species to one species per family to calculate how often the *n* sample size was outside one standard deviation of the overall mean. Based on this process, the minimum number of species required to represent families accurately (within 5% of the overall mean) was consistently nine [25]. Accordingly, nine species were randomly selected for every family except for families containing nine or fewer species, for which all species with available data were used. If a particular datum (body mass, clutch size or egg dimensions) could not be determined for a selected species, a subsequent random selection was made.

Applying this approach, we collected body mass, egg dimension and clutch size data for 1,364 randomly-selected species representing 662 genera from 204 families across all 30 orders of modern birds (means and standard deviations summarized in S1 Table). External length and maximum breadth of eggs (L and B, respectively; in mm) were collected primarily from Schönwetter’s monograph [26] with additional data taken from the primary literature and museum specimens (see [25] for complete list of data sources) and used to calculate egg volume (V) using Hoyt’s [27] formula: *V* = 0.509 *LB* ^*2*^. While other formulae include asymmetry and bicone to improve accuracy [28], [29], this equation determines the volume of all but the most highly pyriform eggs to within 2% of the actual volume measured directly by displacement and, as such, is ideally suited to higher-level comparisons [30], [31]. Clutch size data were collected from multiple sources including books, refereed journals and museum specimen data. Where possible, modal clutch size for each species was collected; if unavailable, mean values were used. The only taxa for which a clutch couldn’t be defined were brood-parasites so they were not included in clutch volume analyses (after [32]). Body mass data were obtained primarily from [33] with further data acquired from other published sources and specimen data. Body mass (in grams) for females was preferentially collected but, if unavailable, male or unknown gender body mass data were used. Where possible, actual values of body mass for each species were used, but when sources quoted a range, the midpoint was used (after [34]). There were insufficient data (*i*.*e*., body mass, clutch size and/or egg dimensions undescribed or otherwise unavailable) to calculate reliable means for seven families (all within the Passeriformes): Conopophagidae, Oxyruncidae, Rhabdornithidae, Melanocharitidae, Paramythiidae, Pityriaseidae and Viduidae (S1 Table).

### Analyses

Least squares linear regression was used to investigate the relationship between body mass and reproductive investment—both egg volume and clutch volume (after [17], [35], [21]). The sum of squares Type III was used, with parameter estimates included in the outputs. A QQ plot revealed the distribution of errors departed from normality, and was corrected using natural log transformation of both clutch volume and body mass (after [36], [37]). These regressions were performed using each of clutch size, egg volume, or clutch volume in turn as the dependent variable, and body mass as the independent variable using the phylogenetic analysis program CAIC [38] and two different phylogenies to ensure that the results were not dependent on the topography of a single phylogenetic hypothesis. Initially we used the family level avian phylogeny of Sibley & Ahlquist [39], which is based on DNA—DNA hybridization and until recently was the only class-wide phylogeny for the birds. Although outdated, this phylogeny was the basis for all phylogenetically-corrected comparisons of avian evolutionary ecology until recently, facilitating comparions between our findings and previous work (*e*.*g*., [24]). We also combined two class-wide avian phylogenies [40], [41], which together provide an updated evolutionary hypothesis for the relationships between avian families. Because these phylogenies were based on different arrangements of families, species were re-arranged and family-level values recalculated where appropriate, for each analysis. Since the Sibley & Ahlquist phylogeny includes branch length information, while the Barker & Cracraft phylogeny does not, we performed all analyses with three phylogenetic hypotheses: Sibley & Ahlquist with branch length information, the same topology but with assumed equal branch lengths, and finally the Barker & Cracraft phylogeny with assumed equal branch lengths. Although yielding qualitatively different results, all phylogenetically-corrected analyses of egg and clutch volume were comparable with the uncorrected data and the same taxa were consistently indentified as outliers. The two plots presented here are for the uncorrected data, with additional plots (Figure A in S1 File) and associated phylogenetically-corrected test statistics (Tables A, B and C in S1 File) and taxa with large residuals summarized in the supplementary files.

To evaluate within-family variability, body mass was divided by clutch volume for each species within a family and the resultant mean and standard deviation used to compute a co-efficient of variation for each family. While these values ranged from 2.5% to 87.9%, 80.2% of the values were less than 50%, demonstrating that the family mean accurately and consistently represented the variation within the family [25]. The overall data set was checked for heteroscedasticity by plotting the co-efficient of variation as a function of the number of species within the family, with no relationship detected (r^2^ = 0.0658, P = 0.349), indicating larger families were not inherently more variable in body mass or clutch volume.

## Results

Body mass and clutch volume ranged over four orders of magnitude, from the 2.6 g *Doricha enicura* with a clutch volume of 0.74 ml to the 83,500 g *Struthio camelus* with a clutch volume of 9,700 ml.

**Fig 1:**
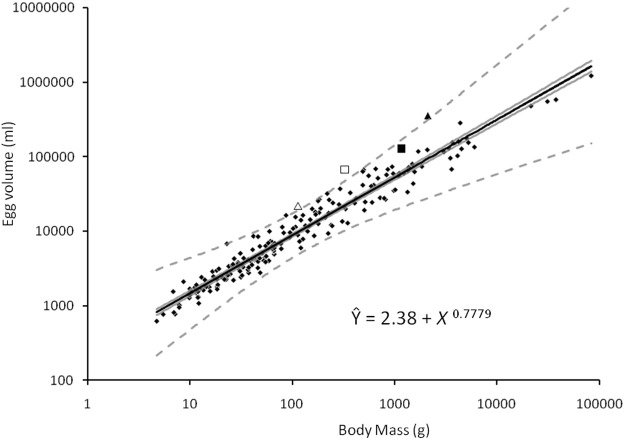
Family means of egg volume expressed as a function of mean body mass (families follow Dickinson 2003), grey lines denoting confidence intervals (standard error) and dotted lines defining 95% confidence intervals. Three families produce an egg more than two standard deviations greater than the volume predicted by body size: Pelecanoidiidae (Diving Petrels; denoted as an open triangle), Dromadidae (Crab Plover, denoted as an open square) and Apterygidae (Kiwis; denoted as a closed triangle); Megapodiidae (Megapodes; denoted as a closed square) not departing from the overall relationship.

There was no significant relationship (p < 0.001) between body mass and clutch size, either with the uncorrected family means or when analyses were corrected for phylogeny (Table A in S1 File). The relationship between body mass and egg volume (Fig. 1) was highly significant (n = 204, r^2^ = 0.951, p<0.001), the pattern and strength of relationship persisting when we controlled for phylogeny using two other family arrangements (99<n<134, 0.790< r^2^< 0.906, 493.46<F<969.27, p<0.001; Table S2). Expressing the regression line as a predictive equation, Ŷ = 2.38 + *X* ^0.7779^ where Y is log (egg volume) and X is log (body mass). Taxa with large residuals included Anhingidae, which had small egg volumes for their body mass, and **Apterygidae**, **Pelecanoidiididae**, **Sternidae**, **Dromadidiae**, Leptosomatidae and Paramythiidae all of which had larger egg volumes than predicted by their body mass (those families with values more than two standard deviations from the mean denoted in bold).

**Fig 2:**
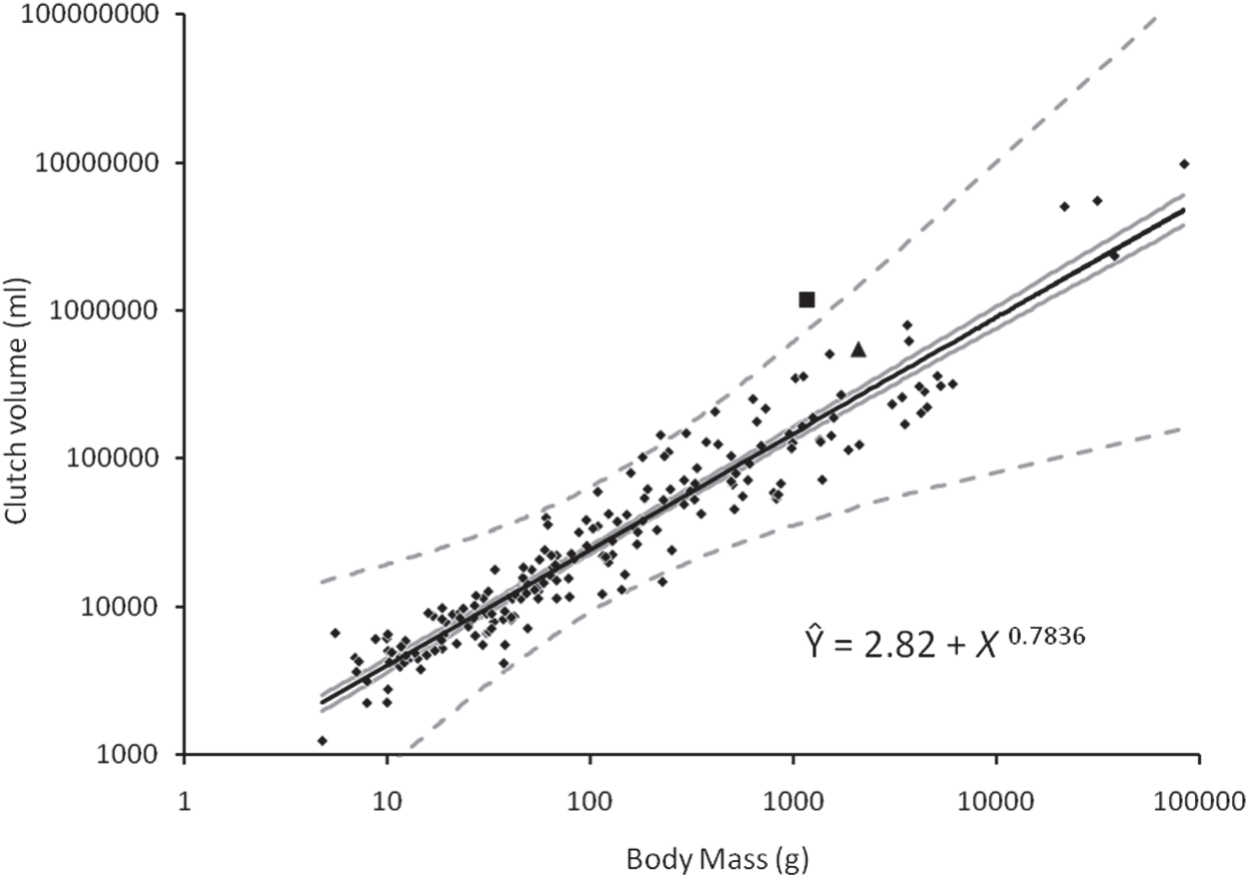
Family means of clutch volume in relation to the mean body mass for avian families (families follow Dickinson 2003), grey lines denoting confidence intervals (standard error) and dotted lines defining 95% confidence intervals. Megapodes (denoted by a square) represent the only outlier with a clutch volume more than 10 standard deviations greater than the value predicted by body mass. The clutch volume of Kiwis (Apterygidae; denoted by a triangle) is not significantly greater than the value predicted by body mass.

The relationship between body mass and clutch volume (Fig. 2) was highly significant (n = 204, r^2^ = 0.9088, p<0.001), regardless of family arrangements and phylogenetic structure (102<n<129, 0.700<r^2^<0.840, 313.72<F<529.36, p<0.001; Table S3). Taxa with large residuals included **Columbidae**, which had small clutch volumes for their body mass and Rheidae, Scolopacidae Crotophagidae, **Leptosomatidae** and Regulidae all of which had large clutch volumes for their body mass (those families with values more than two standard deviations from the mean denoted in bold). Expressing the regression line as a predictive equation, Ŷ = 2.82 + *X* ^0.7836^ where Y is log (clutch volume) and X is log (body mass). The only family to depart significantly from this relationship was Megapodiidae, for which clutch volume was more than ten standard deviations greater than predicted by their body mass.

To explore the basis of the highly divergent value for megapodes, observed values of egg volume and clutch volume for megapodes and all other families of the Galliformes were compared with values predicted by body mass (Table 1). Although all families exhibited greater clutch volumes than those values predicted by body mass, all but two families exhibited clutch volumes within two standard deviations of the value predicted from body mass. Despite having smaller eggs than predicted by body mass, the large clutch size of turkeys (Meleagrididae; mean of 11.8) resulted in a clutch volume almost double the predicted value. The mean value for Megapodiidae egg volume was greater than twice the value predicted by body mass and their mean clutch volume was 7.1 times the predicted value. So, although Megapodiidae were comparable to Numididae (guineafowl) in body mass (1.16 kg and 1.02 kg, respectively) and clutch size (9.6 and 9.8, respectively) the eggs were 3.59 times larger and the overall clutch volume 3.41 times greater. Expressing the equation on the scale of the response variable, the body mass of birds laying a clutch of megapode eggs was predicted to be 8.85 kg (7.6 times their actual mean mass; comparable to the Pelicanidae).

**Table 1:** 

Family	Body Mass (kg)	Clutch size	Egg volume (l)		Clutch volume (l)	
			Observed	Predicted	Observed	Predicted
Megapodiidae	1.16 (0.57)	9.6 (2.6)	0.13 (0.039)	**0.0592 (0.034)**	1.18 (0.49)	**0.167 (0.096)**
Cracidae	1.70 (0.90)	2.4 (0.5)	0.12 (0.041)	0.080 (0.049)	0.27 (0.068)	0.23 (0.14)
Meleagrididae	3.61 (0.86)	11.8 (0.4)	0.068 (0.0030)	0.14 (0.047)	0.79 (0.011)	**0.41 (0.13)**
Tetraonidae	0.72 (0.46)	7.6 (2.4)	0.028 (0.010)	0.041 (0.029)	0.22 (0.10)	0.12 (0.080)
Odontophoridae	0.24 (0.093)	9.6 (4.5)	0.012 (0.0025)	0.017 (0.0083)	0.11 (0.032)	0.048 (0.023)
Phasianidae	0.408 (0.25)	7.6 (6.2)	0.024 (0.0096)	0.026 (0.018)	0.21 (0.26)	0.073 (0.050)
Numididae	1.02 (0.25)	9.8 (3.6)	0.036 (0.0080)	0.053 (0.018)	0.35 (0.15)	0.15 (0.049)

Family means and standard deviations for all families in the Galliformes, with predictions for egg volume and clutch volume based on body mass (bold denotes those predictions greater or less than two standard deviations from the observed mean value).

## Discussion

We confirmed the very close relationship between egg volume and body mass across the class Aves, with body mass explaining about 95% of variability in egg volume at the family scale (79–90.6% after correcting for phylogeny). The relationship was surprisingly similar to Rahn *et al’s* (1975) analysis (Ŷ = 6.5071 + X^0.7817^ and Ŷ = 2.38 + *X* ^0.7779^ respectively), despite using different methods (egg mass vs egg volume), different effective sample sizes (809 species vs 99–204 families, depending on the arrangement used) and expanded taxonomic coverage (they used 809 species from 16 families compared to 1,364 species from 204 families. Similarly, the taxa with large residuals in the egg volume analysis (Fig. 1; Table B in S1 File) equate to those groups previously identified as having disproportionately large eggs relative to their body mass.

Having confirmed the close relationship between egg volume and body mass across the class Aves, a different picture emerged when reproductive investment was considered in terms of clutch volume. Clutch volume was accurately predicted by body mass (within two standard deviations) for all but one of the taxa with disproportionately large individual eggs. An unexpected result was that the plotted value for the Apterygidae (kiwis) was very close to the regression line. This family has long been considered the highlight of extreme avian reproduction allometry. While the volume of a single egg relative to the size of a kiwi is indeed astonishing, their clutch size is small (range of 1 to 2, family mean of 1.49), so their overall clutch volume is not extraordinary. While various mechanisms proposed by previous authors to account for the reproductive strategy of kiwis may still be valid [42], [43], they can no longer be considered to invest more in overall reproduction than expected. Rather, the family displaying the largest clutch volumed relative to body mass was the Megapodiidae (mound-builders or megapodes; Fig. 2). Although comparable to Numididae (guineafowl) in body mass and clutch size, individual megapode eggs are almost four times the volume, yielding the equivalent clutch volume of birds more than seven times their mass.

We suggest that the ultimate factor explaining the disproportionately large reproductive investment in megapodes is their incubation strategy, consistent with Lack’s [11] explanation for their large clutch size. Unlike all other birds, megapodes do not use body heat for incubation, relying instead on environmental heat to warm their eggs. Some species dig burrows near geothermal vents or in sun-warmed sand, others build elaborate mounds of decomposing vegetation matter and insulating sand layers, with constant attendance by the male maintaining optimal incubation temperatures [44]. Megapode eggs have disproportionately large yolks and thin shells [45] covered with calcite nanospheres [46], characteristics considered to enhance gas exchange [47], facilitate hatching [44] and prevent microbial incursion. Being freed from brooding their eggs via body heat, clutch sizes and egg volumes can increase, the larger eggs accommodating larger yolks which allow the chicks to be independent as soon as they hatch [44]. Megapodes are generally considered to be basal within the Galliformes [48] (and references therein) so our results suggest their large clutch size is plesiomorphic, increasing egg size evolving in association with their alternative incubation strategy. Given that only one group has evolved an alternative to regular incubation (and all species within that group) further comparative analysis cannot yield further insight into the role of incubation strategy in determining clutch sizes in birds. Rather, other amniotes such as snakes and lizards may be more suited for these comparisons, with their greater variation in reproductive strategies allowing a more quantitative comparative approach [49], [50].

Rather than just explaining one anomalous family, however, this hypothesis may be fundamental to the explanation of the limitation of clutch size in birds—is clutch size ultimately determined by how many eggs can be brooded? The only other birds that are freed from incubation are obligate brood parasites—although still requiring body heat to incubate the eggs, they rely on the body heat of their hosts. While their eggs tend to be small for their body mass to mimic their hosts’ eggs [51] they can lay many more eggs, thus greatly increasing their clutch volume. Hence, available data are consistent with the hypothesis that the upper limit of clutch size in birds may be constrained by how many eggs can be incubated, rather than the number of eggs which can be laid [9] (but see [52]).

As several researchers have noted, research to-date on avian reproductive investment and life history generally has been dominated by clutch size [14] and it has been unclear if correlates of clutch size were idiosyncratic or reflected determinants of reproductive effort generally. We have confirmed that clutch volume is an easily derived and biologically meaningful measure of reproductive investment, revealing several hitherto overlooked patterns and yielding fresh insight into factors constraining avian reproductive output. Having established the overall patterns of clutch volume in birds and identified those taxonomic and ecological groups that diverge from the norm, many questions arise regarding the evolution and maintenance of reproductive strategies in birds. How do patterns in extant birds compare with extinct groups and fossil taxa? Unlike many other measures of reproductive investment, egg volume can be readily estimated from fossils, enabling comparisons between modern taxa and the very largest birds and theropod dinosaurs. Was there a shift in reproductive strategy associated with the evolution of flight, and do we see comparable patterns in insects?

In addition to these higher-level comparisons, our work stimulates a range of questions at the within family scale. Do changes in clutch size within families, genera and species necessarily involve laying additional eggs, or are they simply redistributing the same investment in a different number of eggs [53]? To maximise the value of these finer-scale studies within lineages, we advocate specifically targeting those groups for which resolved species-level phylogenies have been developed and that exhibit broad variation in the character traits of interest. Thus, Furnariidae for nest type, Pachycephalide for sexual dichromatism and Accipitridae for sexual size dimorphism could provide useful models for further comparative analysis. Having discovered the strong relationship across the entire class, this detailed work will help unveil the exceptions and subtleties, thereby enhancing our understanding of the combined roles of ecology and evolution in sculpting patterns of avian reproduction.

We recognize that the energy associated with egg-laying and incubation represents a subset of the net investment in parental care, with different groups of birds exhibiting wide variation in parental care [12], [9]. Indeed, recent work by Wojczulanis-Jakubas *et al*. [54] suggests the energetic investment in egg production may be lower than previously assumed, with energetic costs associated with incubation in female birds comparable to mate-guarding and parental care by male birds. Thus, our finding that clutch volume scales so closely with body mass suggests that further work should focus on which set of life history traits best explain departures from this overall relationship. Given the minimal post-incubation parental care exhibited by megapodes compared with some groups that provide long-term post-fledging parental care, comparison of overall reproductive costs across birds may reveal far less variation in lifetime reproductive investment.

## Supporting Information

S1 File Supporting information. Table A, Summary of tests estimating the relationship between body mass and clutch size (uncorrected tests and phylogenetically-corrected regressions using CAIC) with two family arrangements. Table B, Summary of tests estimating the relationship between body mass and egg volume (uncorrected tests and phylogenetically-corrected regressions using CAIC) with two family arrangements. Table C, Summary of tests estimating the relationship between body mass and clutch volume (uncorrected tests and phylogenetically-corrected regressions using CAIC) with two family arrangements. Figure A, Results of phylogenetic regression analysis of the relationship between mean clutch size (top row), egg volume (middle row), and clutch volume (bottom row) and mean body mass for avian families. Analysis using the Cracraft-Barker phylogeny (which had no branch length data) appears in the left-hand column, that using the Sibley-Monroe phylogeny without its associated branch length data is in the centre column, and that using the same phylogeny, but with branch lengths, is in the right-hand column. Outliers are not shown in the plots, but are described in the text.
(DOCX)

S1 Table Summary of family means and variance estimates (standard deviations) for body mass, clutch size, egg volume and clutch volume. Family arrangement follows Dickinson (2003).
(XLS)

## References

[1] Pianka ER (1970) On r and K selection. Am Nat 104: 592–597.

[2] Smith CC, Fretwell SD (1974) The optimal balance between size and number of offspring. Am Nat 108: 499–506.

[3] Parker GA, Begon M (1986) Optimal egg size and clutch size: effects of environmental and maternal phenotype. Am Nat 128: 573–592.

[4] Bernardo J (1996) The particular maternal effect of propagule size, especially egg size: patterns, models, quality of evidence and interpretations. Amer Zool 36: 216–236.

[5] Blueweiss L, Fox H, Kudzma V, Nakashima D, Peters R, et al (1978) Relationships between body size and some life history parameters. Oecol 37: 257–272. 10.1007/BF00344996 28309655

[6] Ford NB, Seigel RA (1989) Relationship among body size, clutch size, and egg size in three species of oviparous snakes. Herpetologica 45: 75–83.

[7] Morrison C, Hero J-M (2003) Geographic variation in life-history characteristics of amphibians: a review. J Anim Ecol 72: 270–279.

[8] Lovich JE, Madrak SV, Drost CA, Monatesti AJ, Casper D, et al (2012) Optimal egg size in a suboptimal environment: reproductive ecology of female Sonora mud turtles (*Kinosternon sonoriense*) in central Arizona, USA. Amphibia-Reptilia 33: 161–170.

[9] Ligon JD (1999) The evolution of avian breeding systems. Oxford University Press, Oxford, UK 504 p.

[10] Moreau RE (1944) Clutch size: a comparative study, with reference to African birds. Ibis 86: 286–347.

[11] Lack D (1947) The significance of clutch-size. I and II. Ibis 89: 302–352.

[12] Lack D (1948) The significance of clutch size. III. Some interspecific comparisons. Ibis 90: 25–45.

[13] Slagsvold T (1981) Clutch size and population stability in birds: a test of hypotheses. Oecol 49: 213–217. 10.1007/BF00349190 28309311

[14] Martin TE (2004) Avian life-history evolution has an eminent past: does it have a bright future? Auk 121: 289–301.

[15] Martin TE, Bassar RD, Bassar SK, Fontaine JJ, Lloyd P, et al (2006) Life-history and ecological correlates of geographic variation in egg and clutch mass among passerine species. Evolution 60: 390–398. 16610329

[16] Heinroth O (1922) Die beziehungen zwischen vogelgewicht, eigewicht, gelegegewicht und brutdauer. J Fur Orn 70: 172–285.

[17] Rahn H, Paganelli CV, Ar A (1975) Relation of avian egg weight to body weight. Auk 92: 750–765.

[18] Wiebe KL, Bortolotti GR (1995) Egg size and clutch size in the reproductive investment of American kestrel. J Zool 237: 285–301.

[19] Sæther BE (1987) The influence of body weight on the covariation between reproductive traits in European birds. Oikos 48: 79–88.

[20] Rafferty NE, Boersma PD, Rebstock GA (2005) Intraclutch egg-size variation in Magellanic Penguins. Condor 107: 923–928.

[21] Olsen PD, Cunningham RB, Donnelly CF (1994)a Avian egg morphometrics: allometric models of egg volume, clutch volume and shape. Aust J Zool 42: 307–312.

[22] Dickinson E (2003) The Howard and Moore complete checklist of the birds of the world, 3rd Edition London: Christopher Helm 1040 p.

[23] Owens IPF, Bennett PM (1995) Ancient ecological diversification explains life-history variation among living birds. Proc R Soc Lond B 261: 227–232.

[24] Bennett PM, Owens IPF (2002) Evolutionary ecology of birds: life histories, mating systems and extinction. Oxford University Press, Oxford 278 p.

[25] Anderson SA (2002) The relationship between clutch volume and body mass in birds. Honours thesis. Charles Sturt University.

[26] Schönwetter M (1960–1988) Handbuch der oologie (herau sgegeben Von Dr Wilhelm Meise) Zoologisches Staatsininstitut und Zoologisches Museum Hamburg. Akademie-Verlag, Berlin.

[27] Hoyt DF (1979) Practical methods for estimating volume and fresh weight of bird eggs. Auk 96: 73–77.

[28] Hoyt DF (1976) The effect of shape on the surface-volume relationship of birds’ eggs. Condor 78: 343–349.

[29] Preston FW (1974) The volume of an egg. Auk 91: 132–138.

[30] Potti J (1999) Maternal effects and the pervasive impact of nestling history in egg size in a passerine bird. Evol 53: 279–285. 10.1111/j.1558-5646.1999.tb05353.x 28565183

[31] Ramos JA (2001) Seasonal variation in reproductive measures of tropical Roseate Terns *Sterna dougallii*: previously undescribed breeding patterns in a seabird. Ibis 143: 83–91.

[32] Jetz W, Sekercioglu CH, Böhning-Gaese K (2008) The worldwide variation in avian clutch size across species and space. PLoS Biol 6: 2650–2657. 10.1371/journal.pbio.0060303 19071959 PMC2596859

[33] Dunning JB (2008) CRC handbook of avian body masses. 2nd Edition CRC Press, London 655 pp.

[34] Brooke MD, Hanley S, Laughlin SB (1999) The scaling of eye size with body mass in birds. Proc. Royal Soc. Lond B 266: 405–412.

[35] Seim E (1983) On rethinking allometry: which regression model to use? J Theor Biol 104: 161–168.

[36] Nealen PM, Ricklefs RE (2001) Early diversification of the avian brain: body relationship. J Zool London 253: 391–404.

[37] Rayner JMV (1985) Linear relations in biomechanics: the statistics of scaling functions. J Zool London 206: 415–439.

[38] Purvis A, Rambaut A (1995) Comparative analysis by independent contrasts (CAIC): an Apple Macintosh application for analysing comparative data. Comput Appl Biosci 11: 247–251. 7583692 10.1093/bioinformatics/11.3.247

[39] Sibley CG, Ahlquist JE (1990) Phylogeny and classification of birds. Yale University Press, New Haven, Connecticut 976 p.

[40] Barker FK, Cibois A, Schikler PA, Feinstein J, Cracraft J (2004) Phylogeny and diversification of the largest avian radiation. Proc Nat Acad Sci USA 101:11040–11045. 15263073 10.1073/pnas.0401892101 PMC503738

[41] Cracraft J, Barker FK, Braun M, Harshman J, Dyke GJ, et al (2004) Phylogenetic relationships among modern birds (Neornithes): towards an avian tree of life In: Cracraft J, Donoghue MJ, editors. Assembling the Tree of Life. Oxford, GB, Oxford University Press pp. 468–489

[42] Cracraft J (1974) Phylogeny and evolution of the ratite birds. Ibis 116: 494–521.

[43] Calder WA (1979) The kiwi and egg design: evolution as a package deal. BioScience 29:461–467.

[44] Jones DN, Dekker RWRJ, Roselaar CS (1995) The Megapodes (Megapodiidae). Oxford University Press, Oxford 262 p

[45] Booth DT (1988) Shell thickness in megapode eggs. Megapode Newsletter 2: 13.

[46] D’Alba L, Jones DN, Badaway HT, Eliason CM, Shawkey MD (2014) Antimicrobial properties of nanostructured eggshell from a compost-nesting bird. J Exp Biol 217: 1116–1121. 10.1242/jeb.098343 24311808

[47] Christensen VL, Davis GS, Lucore LA (1996) Eggshell conductance and other functional qualities of ostrich eggs. Poultry Sci 75: 1404–1410. 8933594 10.3382/ps.0751404

[48] Wang N, Kimball RT, Braun EL, Liang B, Zhang Z (2013) Assessing phylogenetic relationships among Galliformes: a multigene phylogeny with expanded taxon sampling in Phasianidae. PLoS ONE 8(5): e64312 10.1371/journal.pone.0064312 23741315 PMC3669371

[49] Qualls SP, Shine R (1995) Maternal body volume as a constraint on reproductive output in lizards: evidence from the evolution of viviparity. Oecol 103: 73–78. 10.1007/BF00328427 28306947

[50] Spencer RJ, Thompson MB, Banks PB (2001) Hatch or wait? A dilemma in reptilian incubation. Oikos 93: 401–406.

[51] Johnsgard PA (1997) The avian brood parasites: deception at the nest. Oxford University Press, Oxford 409 p

[52] Klomp H (1970) The determination of clutch-size in birds: a review. Ardea 58: 1–124.

[53] Olsen PD, Cunningham RB, Donnelly CF (1994)b Is there a trade-off between egg size and clutch size in altricial and precocial non-passerines? A test of a model of the relationship between egg and clutch size. Aust J Zool 42: 323–328.

[54] Wojczulanis-Jakubas K, Jakubas D, Kulaszewicz I, Kidawa D, Taylor JRE (2014) Influence of primary reproductive investments on blood biochemistry, leukocyte profile, and body mass in a small Arctic seabird. Auk 131: 743–755. 10.1111/jnc.12970 25319762

