# Supplementary material for: Reassessing Breeding Investment in Birds: Class-Wide Analysis of Clutch Volume Reveals a Single Outlying Family
Source: PLoS One. 2015 Jan 29;10(1):e0117678. doi: 10.1371/journal.pone.0117678 (PMC4310587; doi:10.1371/journal.pone.0117678)

**Supplementary Information**

**“Reassessing breeding investment in birds: class-wide analysis of clutch volume reveals a single outlying family” by David M Watson, Susan Anderson and Valerie Olson**

**Table A. Summary of tests estimating the relationship between body mass and clutch size (uncorrected tests and phylogenetically-corrected regressions using CAIC) with two family arrangements.**

| Tests using Sibley-Monroe family structure | N | Slope | intercept | r-squared | F |
| --- | --- | --- | --- | --- | --- |
| non-phylogenetic (all data) | 141 | 0.379 ± 0.201 | 2.468 ± 0.459 | 0.025 | 3.559 |
| non-phylogenetic (outliers removed) | 136 | 0.213 ± 0.173 | 2.601 ± 0.391 | 0.010 | 1.517 |
| phylogenetic (no branch lengths) | 134 | -0.282 ± 0.306 | NA | 0.010 | 0.870 |
| phylogenetic (no branch lengths; outliers removed) | 128 | -0.199 ± 0.224 | NA | 0.010 | 0.790 |
| phylogenetic (branch lengths) | 134 | 0.673 ± 0.318 | NA | 0.030 | 4.460* |
| phylogenetic (branch lengths, outliers removed) | 126 | 0.198 ± 0.182 | NA | 0.010 | 1.190 |
|  |  |  |  |  |  |
| Tests using Cracraft-Barker family structure |  |  |  |  |  |
| non-phylogenetic (all data) | 137 | 0.322 ± 0.180 | 2.520 ± 0.412 | 0.023 | 3.187 |
| non-phylogenetic (outliers removed) | 133 | 0.055 ± 0.169 | 2.920 ± 0.376 | 0.001 | 1.450 |
| phylogenetic (no branch lengths) | 107 | -0.360 ± 0.299 | NA | 0.010 | 0.870 |
| phylogenetic (no branch lengths; outliers removed) | 99 | -0.539 ± 0.188 | NA | 0.077 | 8.195** |

* p < 0.05, ** p < 0.01, ***, p < 0.001

Using the Sibley-Monroe family structure, taxa with large residuals in the non-phylogenetic test included Anatidae, Crotophagidae, Dendrocygnidae, Regulidae, and Rheidae, all of which had large clutches for their body mass. Phylogenetic analysis yielded nodal outliers roughly corresponding to these and other groups. Unusual changes in clutch size (for the respective changes in body mass) occurred at the following splits:

1. between the Struthionidae/Rheidae group and the Casuariidae/Apterygidae group
2. between Casuariidae and Apterygidae
3. between Cracidae and Megapodiidae
4. between Phasianidae and the Anseranatidae/Dendrocygnidae group
5. between Coliidae and the cuckoo families
6. between Regulidae and the Pycnonotidae/Cisticolidae/Zosteropidae group

Using the Cracraft-Barker family structure, taxa with large residuals in the non-phylogenetic test included Anatidae, Regulidae, Rheidae, and Struthionidae, all of which had large clutches for their body mass. In the phylogenetic analysis, unusual changes in clutch size (relative to the change in body mass), occurred at the following splits:

1. among families in the Struthioniformes
2. between Struthioniformes and all other birds
3. between the Anseranatidae/Anatidae group and Anhimidae
4. between Megapodiidae and the Cracidae/Galliformes group
5. between Cracidae and all Galliformes
6. between Aramidae and Gruidae
7. between Upupidae and Phoeniculidae
8. between Regulidae and the Bombycillidae/Muscicapidae/Cinclidae/Sturnidae group

Unlike the Sibley-Monroe arrangement of families where no clear relationship between body mass and clutch size was found, a negative relationship between body mass and clutch size is evident using the Cracraft-Barker, but only when controlling for the relationships among families.

**Table B. Summary of tests estimating the relationship between body mass and egg volume (uncorrected tests and phylogenetically-corrected regressions using CAIC) with two family arrangements.**

| Test Using Sibley-Monroe family structure | N | slope | intercept | r-squared | F |
| --- | --- | --- | --- | --- | --- |
| non-phylogenetic (all data) | 141 | 0.764 ± 0.016 | 2.460 ± 0.036 | 0.943 | 2304.00*** |
| non-phylogenetic (outliers removed) | 138 | 0.765 ± 0.015 | 2.453 ± 0.033 | 0.953 | 2776.87*** |
| phylogenetic (no branch lengths) | 134 | 0.643 ± 0.029 | NA | 0.790 | 498.06*** |
| phylogenetic (no branch lengths; outliers removed) | 130 | 0.689 ± 0.025 | NA | 0.850 | 753.51*** |
| phylogenetic (branch lengths) | 134 | 0.625 ± 0.028 | NA | 0.790 | 493.46*** |
| phylogenetic (branch lengths, outliers removed) | 129 | 0.710 ± 0.024 | NA | 0.871 | 864.51*** |
|  |  |  |  |  |  |
| Tests using Cracraft-Barker family structure |  |  |  |  |  |
| non-phylogenetic (all data) | 137 | 0.775 ± 0.016 | 2.430 ± 0.036 | 0.946 | 2372.60*** |
| non-phylogenetic (outliers removed) | 133 | 0.769 ± 0.014 | 2.433 ± 0.031 | 0.960 | 3134.03*** |
| phylogenetic (no branch lengths) | 107 | 0.691 ± 0.030 | NA | 0.830 | 527.28*** |
| phylogenetic (no branch lengths; outliers removed) | 99 | 0.740 ± 0.024 | NA | 0.906 | 969.27*** |

* p < 0.05, ** p < 0.01, ***, p < 0.001

For the Sibley-Monroe family structure, taxa with large residuals in the non-phylogenetic test included Anhingidae, which had small egg volumes for their body mass, and Procellariidae, and Scolopacidae, both of which had large egg volume for their body mass. In the phylogenetic analysis, unusual changes in egg volume (relative to change in body mass) occurred at the following splits:

1. between Casuariidae and Apterygidae
2. between the Cracidae/Megapodiidae group and the Odontophoridae/Numididae group
3. between the Jacanidae/Rostratulidae group and the Pedionomidae/Thinocoridae/Scolpacidae group
4. between Spheniscidae and Gaviidae

Using the Cracraft-Barker family structure, taxa with large residuals in the non-phylogenetic test included Anhingidae, which had small egg volumes for their body mass, and Apterygidae, Procellariidae, and Scolopacidae, all of which had large egg volumes for their body mass. In the phylogenetic analysis, unusual change in egg volume occurred at the following splits:

1. among the families in Struthioniformes
2. between Cracidae and all Galliformes
3. between Glareolidae and Laridae
4. among Procellariidae, Gaviidae, and Spheniscidae
5. between Aegothelidae and the Trochilidae/Apodidae group

The relationship between body mass and egg volume is significant, even when controlling for phylogenetic relationships. However, because the F and r-squared values are lower for the phylogenetic tests than for the non-phylogenetic tests, a small effect of phylogeny on the relationship can be inferred using both topologies.

**Table C. Summary of tests estimating the relationship between body mass and clutch volume (uncorrected tests and phylogenetically-corrected regressions using CAIC) with two family arrangements.**

| Tests using Sibley-Monroe family structure | N | slope | intercept | r-squared | F |
| --- | --- | --- | --- | --- | --- |
| non-phylogenetic (all data) | 141 | 0.781 ± 0.023 | 2.833 ± 0.054 | 0.888 | 1105.70*** |
| non-phylogenetic (outliers removed) | 138 | 0.771 ± 0.022 | 2.845 ± 0.051 | 0.897 | 1181.94*** |
| phylogenetic (no branch lengths) | 134 | 0.638 ± 0.035 | NA | 0.710 | 326.13*** |
| phylogenetic (no branch lengths; outliers removed) | 127 | 0.656 ± 0.028 | NA | 0.811 | 539.26*** |
| phylogenetic (branch lengths) | 134 | 0.722 ± 0.041 | NA | 0.700 | 313.72*** |
| phylogenetic (branch lengths, outliers removed) | 129 | 0.644 ± 0.031 | NA | 0.774 | 439.00*** |
|  |  |  |  |  |  |
| Tests using Cracraft-Barker family structure |  |  |  |  |  |
| non-phylogenetic (all data) | 137 | 0.781 ± 0.022 | 2.821 ± 0.049 | 0.906 | 1300.90*** |
| non-phylogenetic (outliers removed) | 133 | 0.777 ± 0.021 | 2.823 ± 0.048 | 0.912 | 1352.89*** |
| phylogenetic (no branch lengths) | 107 | 0.646 ± 0.033 | NA | 0.780 | 375.80*** |
| phylogenetic (no branch lengths; outliers removed) | 102 | 0.639 ± 0.028 | NA | 0.840 | 529.36*** |

* p < 0.05, ** p < 0.01, ***, p < 0.001

Using the Sibley-Monroe family arrangement, taxa with large residuals in the non-phylogenetic test included Columbidae, which had small clutch volumes for their body mass, and Crotophagidae, Rheidae, and Scolopacidae, all of which had large clutch volumes for their body mass. In the phylogenetic analysis, unusual change in clutch volume occurred at the following splits:

1. at the root node (where the Struthioniformes, Tinamiformes, Craciformes, Galliformes, and Anseriformes split off from all other avian taxa)
2. between Cracidae and Megapodiidae
3. between between Leptosomiidae and Brachypteraciidae
4. between Coliidae and the cuckoo families
5. between Columbidae and Gruiformes
6. between Climacteridae and Menuridae
7. between Regulidae and the Pycnonotidae/Cisticolidae/Zosteropidae group

Using the Cracraft-Barker family structure, taxa with large residuals in the non-phylogenetic test included Columbidae, which had small clutch volumes for their body mass, and Regulidae, Rheidae, and Scolopacidae, all of which had large clutch volumes for their body mass. In the phylogenetic analysis, unusual change in clutch volume occurred at the following splits:

1. between the Struthioniformes and all other birds
2. between Megapodiidae and the Cracidae/Galliformes group
3. between Aramidae and Gruidae
4. between Columbidae and Pteroclidae
5. between Leptosomiidae and the Coraciidae/Brachypteraciidae group

The relationship between body mass and clutch volume is highly significant, even when controlling for phylogenetic relationships. Since the F and r-squared values are lower for the phylogenetic tests than for the non-phylogenetic tests, a small effect of phylogeny is inferred. As with egg volume, the relationship between body mass and clutch volume is unaffected by which phylogeny is used.

Figure A. Results of phylogenetic regression analysis of the relationship between mean clutch size (top row), egg volume (middle row), and clutch volume (bottom row) and mean body mass for avian families. Analysis using the Cracraft-Barker phylogeny (which had no branch length data) appears in the left-hand column, that using the Sibley-Monroe phylogeny without its associated branch length data is in the centre column, and that using the same phylogeny, but with branch lengths, is in the right-hand column.


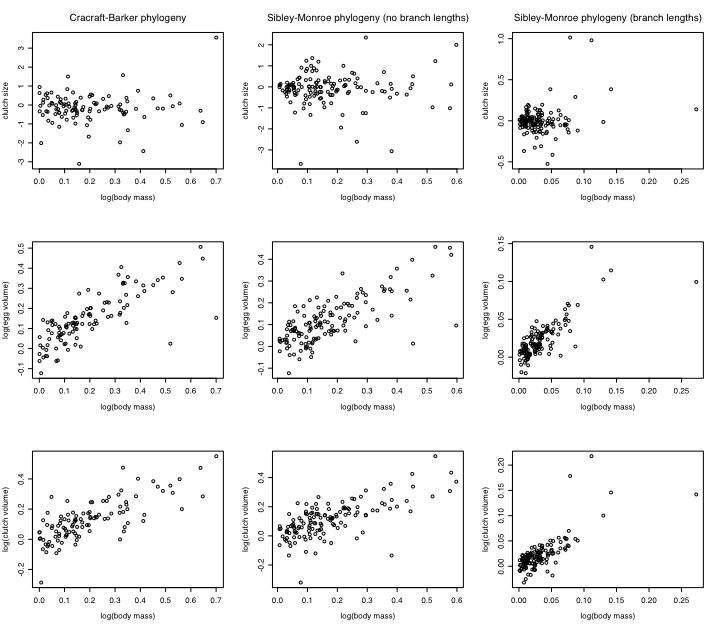

Supplement: S1 File — Table A, Summary of tests estimating the relationship between body mass and clutch size (uncorrected tests and phylogenetically-corrected regressions using CAIC) with two family arrangements. Table B, Summary of tests estimating the relationship between body mass and egg volume (uncorrected tests and phylogenetically-corrected regressions using CAIC) with two family arrangements. Table C, Summary of tests estimating the relationship between body mass and clutch volume (uncorrected tests and phylogenetically-corrected regressions using CAIC) with two family arrangements. Figure A, Results of phylogenetic regression analysis of the relationship between mean clutch size (top row), egg volume (middle row), and clutch volume (bottom row) and mean body mass for avian families. Analysis using the Cracraft-Barker phylogeny (which had no branch length data) appears in the left-hand column, that using the Sibley-Monroe phylogeny without its associated branch length data is in the centre column, and that using the same phylogeny, but with branch lengths, is in the right-hand column. Outliers are not shown in the plots, but are described in the text. (DOCX) [file pone.0117678.s001.docx]
